# Bioethanol Conversion into Propylene over Various Zeolite Catalysts: Reaction Optimization and Catalyst Deactivation

**DOI:** 10.3390/nano12162746

**Published:** 2022-08-10

**Authors:** Wei Xia, Chao Ma, Yaxin Huang, Shuangshuang Li, Xue Wang, Kun Chen, Dong Liu

**Affiliations:** State Key Laboratory of Heavy Oil Processing, College of Chemistry and Chemical Engineering, China University of Petroleum (East China), 66 Changjiang West Road, Huangdao District, Qingdao 266580, China

**Keywords:** ethanol, propylene, zeolites, ZSM-5

## Abstract

Catalytic conversions of bioethanol to propylene were investigated over different zeolite catalysts. H-ZSM-5 (SiO_2_/Al_2_O_3_ = 80) was found to be the most effective for propylene production. Furthermore, H-ZSM-5 (SiO_2_/Al_2_O_3_ = 80) was investigated under different variables of catalytic reaction (calcination temperature, feed composition, reaction temperature, and time on stream) for the conversion of ethanol to propylene. The H-ZSM-5(80) catalysts calcined at 600 °C showed the highest propylene yield. The moderate acidic site on ZSM-5 is required for the production of propylene. The activity on ZSM-5 is independent of the ethanol feed composition. H-ZSM-5 catalyst deactivation was observed, owing to dealumination. The highest propylene yield was 23.4% obtained over HZSM-5(80). Propylene, butene, and ≥C5 olefins were formed by parallel reaction from ethylene. Olefins were converted to each paraffin by sequential hydrogenation reaction. HZSM-5(80) catalyst is a promising catalyst not only for ethanol but also for the conversion of bioethanol to light olefins.

## 1. Introduction

As the carbon neutrality goal approaches, countries around the world are urgently looking for renewable energy alternatives to fossil fuels to achieve their carbon neutrality commitments. Cellulosic ethanol can be derived from crop waste, forestry waste and municipal organic waste, therefore it has attracted much attention as a carbon-neutral energy source [1,2].

Cellulosic ethanol is currently mainly used as a biofuel added to gasoline, but this fuel cannot be called a clean energy due to its low calorific value and instability, as well as the pollutants produced by its use. Therefore, the conversion of cellulose ethanol to olefin (ETO) has become the preferred production route.

Since it was recognized that petroleum resources are not renewable in the 1970s, the process of ethanol dehydration to ethylene has received attention. But increasing ethylene capacity has led researchers to turn their attention to propylene. In the past two decades, the study of the conversion of ethanol to propylene (ETP) has become a research hotspot [3], and some researchers have reported the conversion of ethanol to hydrocarbons over unmodified or metal-modified ZSM-5 catalysts [4,5,6,7,8,9,10,11,12,13,14,15]. Other zeolites [15,16,17] (such as mordenite, beta-zeolite, ferrierite, Y-zeolite, and SAPO-34) have also been used as catalysts for the ETO reaction. Our group has been focusing on ethanol conversion reactions on ZSM-5 or metal oxides. Recently, Hua et al. synthesized Sm-CeO_2_/Beta composites catalyst for ETP. The propylene yield was more than 60% [18,19,20]. All previous studies have shown that zeolites are strong candidates to catalyze the ETO reaction, but the process has not been extensively studied [6,9,10,11,12,13,14,15,16,17,21,22,23,24,25,26].

Different zeolites have different structural characteristics such as pore size, pore volume, acidic sites and strength. These structural and chemical parameters and reaction variables are of critical importance for any catalytic reaction. We studied ETP reactions on different zeolites. For the most effective catalyst, the effects of operating variables (calcination temperature, feed composition, reaction temperature, and contact time) on the yield of products were further studied in this work. Exploration of these reaction variables is necessary for reactor design and feasibility for industrial process applications.

## 2. Experimental

### 2.1. Preparation of the Different Zeolite Catalysts

Mordenite/H-MOR (SiO_2_/Al_2_O_3_ = 20), β-zeolite/H-BEA (SiO_2_/Al_2_O_3_ = 75), Ferrierite/H-FER (SiO_2_/Al_2_O_3_ = 55), Y-zeolite/H-Y (SiO_2_/Al_2_O_3_ = 5.6), and ZSM-5/H-ZSM-5 (SiO_2_/Al_2_O_3_ = 30, 80, and 280) were obtained from Zeolite International. Before reactions, the zeolite catalyst samples were calcined in air at a heating rate of 4.5 °C/min and kept at 600 °C for 4 h. The resulting catalysts were designated H-MOR(20), H-BEA(75), H-FER(55), H-Y (5.6), H-ZSM-5(30), H-ZSM-5(80), and H-ZSM-5(280).

### 2.2. Characterization

The acidic properties of the ZSM-5 zeolite catalyst were explored by temperature- programmed desorption (TPD) technique. The catalyst samples were placed in quartz tubes and preheated in He gas for 1 h (99.99%, 30 cm^3^/min, 500 °C). The catalyst samples were cooled to 100 °C, and adsorbed NH_3_/He (30 cm^3^/min) at 100 °C for 1 h. They were then purged with He (30 cm^3^/min) for 1 h at the same temperature to remove any NH_3_ physically adsorbed on the sample surface. Details of the NH_3_-TPD measurements and ^27^Al MAS NMR measurements can be found in our previous report [6].

### 2.3. Ethanol Conersion Reaction

The ethanol conversions were performed in a continuous-flow fixed-bed reactor, which was controlled at a temperature of 400–600 °C and 0.1MPa. Before each reaction, the catalyst was pretreated in a N_2_ flow (100 cm^3^/min) at 600 °C for 1 h. The reactant ethanol (99.5%, Wako), without further purification, was introduced into the reactor by a micro peristaltic pump (BT 100-3J, China) (ethanol feed composition of 20, 50 and 80% in the N_2_ stream). The reaction mixture was passed through an evaporator before entering the reactor. The reaction products were monitored online by gas chromatography, a hydrogen flame ionization detector for C_1_–C_4_ hydrocarbons (RT-alumina PLOT, Restek USA), and a Shincarbon ST for N_2_ and H_2_ (Shinwa Chem. Ind. Ltd., Japan). Contact time was defined as W/F, where F is the total flow rate (cm^3^/min) and W is the catalyst weight (g). The reaction of ethylene or a mixture of ethylene and water was also investigated using apparatus and procedures as described above.

## 3. Results

### 3.1. Ethanol Conversion over different Zeolite Catalysts

**Figure 1:**
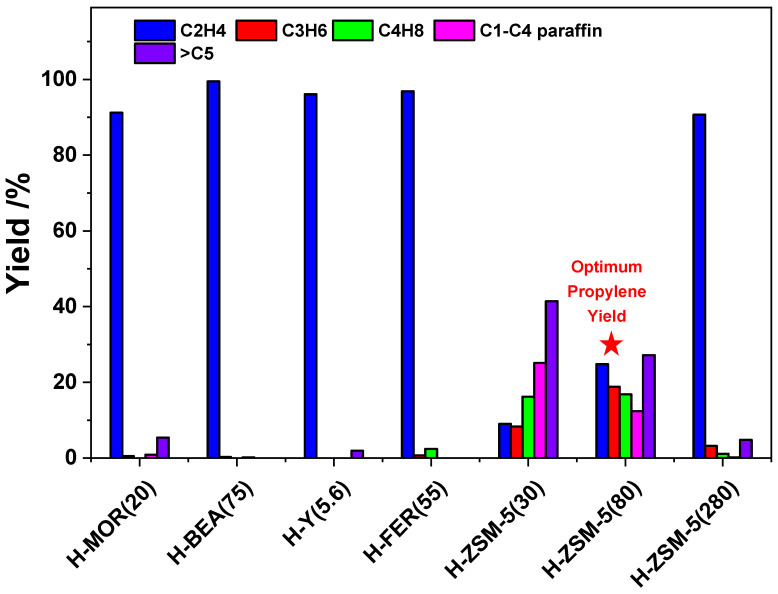
ETO conversion over various zeolites catalysts. Temperature, 400 °C; W/F = 0.0025 g/cm^3^·min^−1^; EtOH concentration, 50%.

Ethanol conversion reactions were explored over different zeolite catalysts at 400 °C under atmospheric pressure. The ethanol conversion and product distributions were presented in Figure 1. Conversions of ethanol to 100% were obtained over H-BEA, H-FER, H-ZSM-5 (30, 80 and 280) catalysts. Ethanol conversions at 98% were obtained over H-MOR and H-Y catalysts. More than 90% ethylene conversion was obtained over H-MOR, H-BEA, H-Y, and H-FER catalysts. The higher propylene yield was obtained over H-ZSM-5 catalysts compared to the other zeolite catalysts. Among these three H-ZSM-5 (30, 80 and 280) catalysts, the highest desired product propylene yield was 18.8% over H-ZSM-5(80). The yields of propylene were in the following order: H-ZSM-5(80) > H-ZSM-5(30) > H-ZSM-5(280). The other products obtained were ethane, propane, C4 (including 1-butene, cis-2-butene, trans-2-butene, isobutylene, butane, and isobutane), and other products (including CH_4_, diethyl ether, C_5+_ aliphatic, and aromatics). Ethanol is therefore most effectively converted into propylene by the ZSM-5(80) catalyst. Moderate surface acid density seems optimum for propylene production.

Comparing these results with previous work, Dias et al. tuned the acidity of MOR, FER and ZSM-5 zeolites by solid-state dealumination for alcohol dehydration. The optimum dealumination degree was explored for each zeolite to enhance catalytic activity for alcohol dehydration [27]. Busica et al. compared H-FER, H-MFI, H-MOR, H-BEA, H-Y and H-USY zeolite, silica alumina, and alumina for ethanol dehydration. Similarly to our results, The H-MOR sample was the most active but the H-MFI samples with SiO_2_/Al_2_O_3_ ratios 280 and 50 showed higher reaction rates [28]. The catalytic performance of different protonic zeolites can be explained by confinement effects on the different zeolite cavities.

### 3.2. Conversion of Ethanol over H-ZSM-5(80) Catalysts

Owing to the optimum performance of H-ZSM-5(80) catalyst for the production of propylene, we investigated the effect of the operating variables (calcination temperature, feed composition, reaction temperature, time on stream, and raw materials) on ethanol and bioethanol conversion reactions.

#### 3.2.1. Calcination Temperature

**Figure 2:**
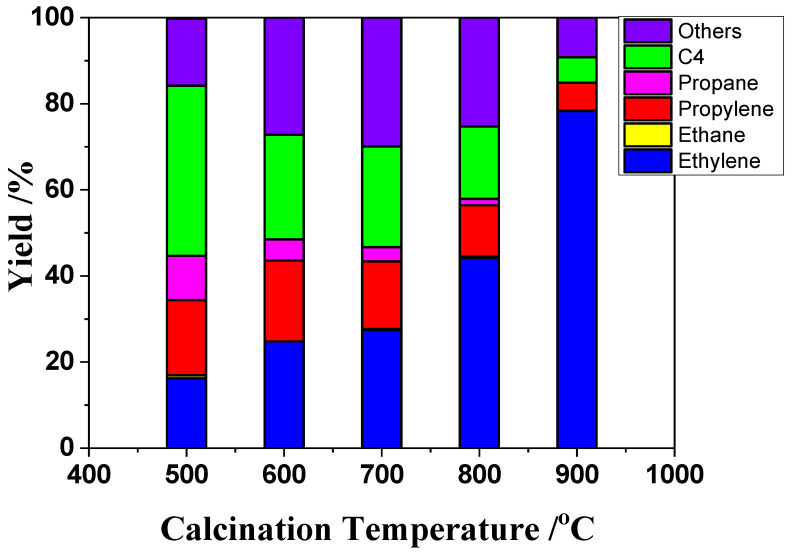
Production distributions over H-ZSM-5(80) catalysts as a function of calcination temperature. Temperature, 400 °C; EtOH concentration, 50%; W/F = 0.0025 g/cm^3^·min^−1^.

The conversion reactions of ETP on a H-ZSM-5(80) catalyst were investigated at different calcination temperatures (500, 600, 700, 800, and 900 °C). As shown in Figure 2, the ethylene yield increased from 16.2 to 78.3% with the calcination temperature increasing from 500 to 900 °C. The propylene yield increased initially, from 17.5 to 18.8%, and then decreased to 6.5% with the calcination temperature increasing from 500 to 900 °C. The H-ZSM-5(80) catalyst calcined at 600 °C showed the highest propylene yield, at 18.8%. We also investigated the NH_3_-TPD data of the H-ZSM-5(80) catalyst. As shown in Figure S1, all the catalyst samples showed similar NH_3_-TPD spectra with two well-resolved NH_3_ desorption peaks: the low-temperature peak at about 170 °C and the high-temperature peak at about 400 °C. Propylene yield decreased, whereas ethylene yield increased with calcination temperatures increasing from 600 to 900 °C. Ethylene was predominantly produced from ethanol over catalysts calcined at 900 °C. As shown in Figure S1, the desorption peaks at 400 °C decreased with calcination temperature increasing from 500 to 900 °C. This acidic site relates to the active sites for production of propylene [20].

#### 3.2.2. Feed Composition

**Figure 3:**
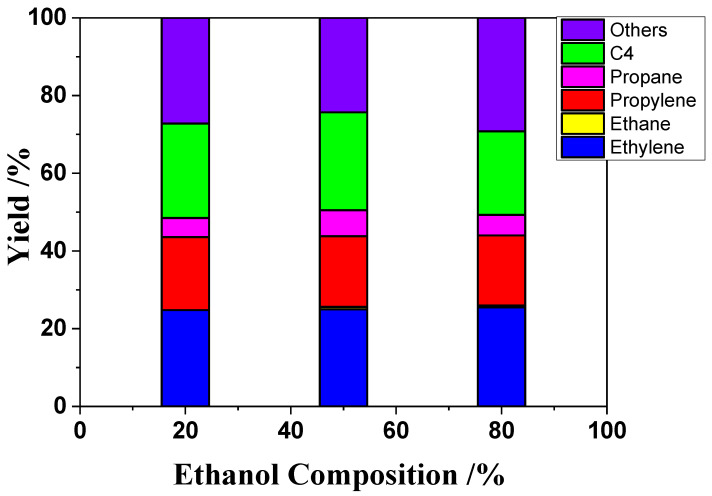
Effect of ethanol composition on ETO reactions over H-ZSM-5(80) catalyst. Temperature, 400 °C; W/F = 0.0025 g/cm^3^·min^−1^.

The effect of ethanol concentration on ETP reactions was examined. Figure 3 shows the ethanol conversions and the yields of product. As shown in Figure 3, the propylene yields remain about 18%, and the yields of ethylene remain almost 25% with ethanol concentration increasing from 20 to 80%. There is almost no difference of production distribution with change of feed composition.

#### 3.2.3. Reaction Temperature

**Figure 4:**
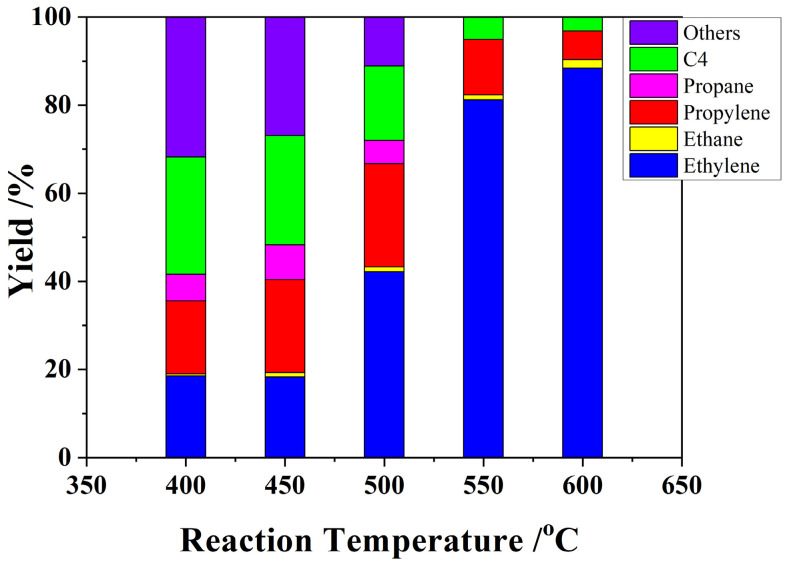
ETO reactions on H-ZSM-5(80) at different temperatures. W/F = 0.0025 g/cm^3^·min^−1^, EtOH concentration, 50%.

The conversions of ETP over H-ZSM-5(80) catalyst were explored from 400 to 600 °C. Figure 4 shows the ethanol conversions and yields to olefins at the different temperatures. The propylene yield increase from 18.8 to 23.4% with increasing reaction temperature from 400 to 500 °C (Figure 4). After that, the propylene yield decreased from 23.4 to 6.5% with the reaction temperature continuously increasing from 500 to 600 °C, whereas the ethylene yield increased from 24.8 to 88.4% with increasing of reaction temperature from 400 to 600 °C. The optimum propylene yield, 23.4%, was obtained at 500 °C. Busca et al. proposed at low temperatures, diethyl ether is the main product of ethanol conversion on H-ZSM-5. When reaction temperature increased to 227–277 °C, ethylene was the main product at full conversion. However, by further increasing reaction temperature, ethylene selectivity dropped in favor of higher hydrocarbons, such as propylene, butenes, butanes, and aromatics [29]. Similarly to our results, ethylene products dominated the product distribution at high reaction temperatures (above 600 °C).

#### 3.2.4. Time on Stream

**Figure 5:**
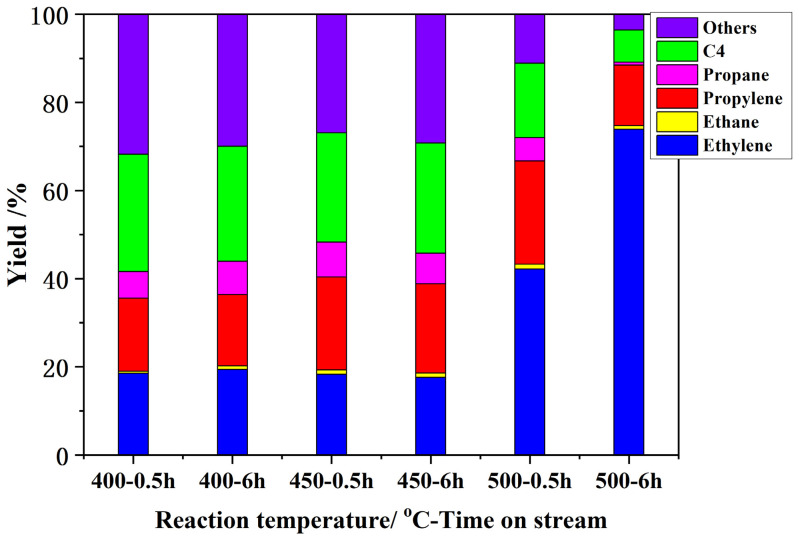
ETO reactions at different temperatures and under different reaction times over H-ZSM-5(80) catalyst. W/F = 0.0025 g/cm^3^·min^−1^, EtOH concentration, 50%.

ETP reaction stability was investigated with time on stream. The conversion of ethanol was maintained at 100% during the experiment (Figure 5). The yields of propylene and other products remained constant at 400 °C. The yields of propylene and other products remained almost constant at a reaction temperature of 450 °C. When the reaction temperature rose to 500 °C, the propylene yield decreased from 23.4 to 13.8%, and ethylene yield increased from 42.2 to 73.9% after 6 h. There was a significant change in the product distribution at 500 °C with time on stream. The maximum yield of propylene was obtained at 500 °C.

**Figure 6:**
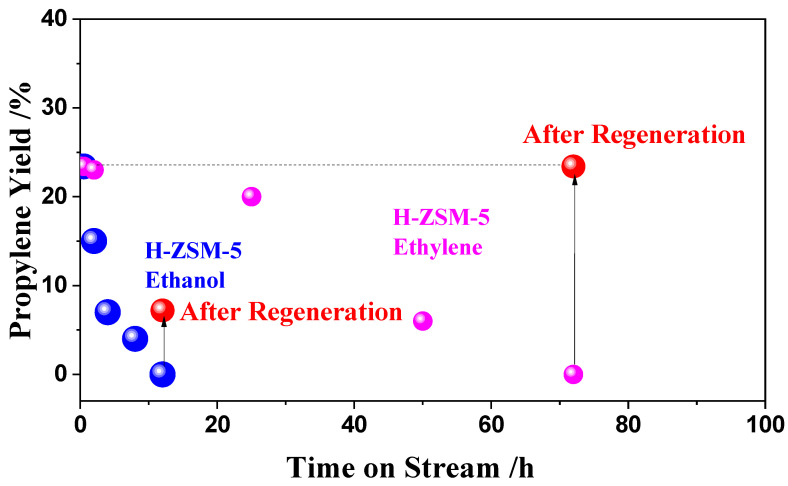
Stability of H-ZSM-5(80) catalysts for ethanol or ethylene conversion. Temperature: 500 °C; W/F = 0.0025 g/cm^3^·min^−1^; Feed composition: ethylene conversion (ethylene: N_2_ = 1:2), ethanol conversion (ethanol: N_2_ = 1:1); regeneration condition: 630 °C for 0.5 h in air.

Since the maximum propylene yield was obtained at 500 °C, the H-ZSM-5(80) catalyst’s stability was further determined by the time course of the propylene yield at 500 °C. As shown in Figure 6, for ethanol conversion, the propylene yield decreased from 23.4% with time on stream, and no propylene was found after 12 h. The initial catalytic activity for the reaction of ethylene was almost 23.4%, which is similar to that for the reaction of ethanol. However, the propylene yield decreased slowly until the yield was zero after 72 h. In the reaction with ethanol as raw material, the yield of propylene decreased much faster than that of ethylene, and the catalyst could only be partially regenerated, while the catalyst with ethylene as raw material could be almost fully regenerated. This shows that the collapse of molecular sieve framework caused by dealumination is the main reason for catalyst deactivation.

**Figure 7:**
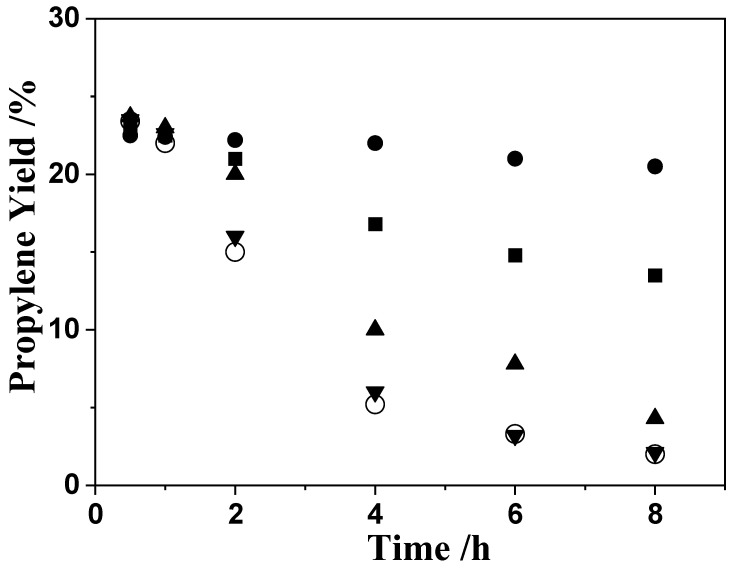
Effects of water on the stability of ETP reactions. Temperature, 500 °C; W/F = 0.0025 g/cm^3^·min^−1^; Feed composition: ● (ethylene: N_2_ = 1: 2), ■ (ethylene: H_2_O: N_2_ = 33: 8: 59), ▲ (ethylene: H_2_O: N_2_ = 33: 17: 50), ▼ (ethylene: H_2_O: N_2_ = 1: 1: 1), ○ (ethanol: N_2_ = 1: 1).

**Figure 8:**
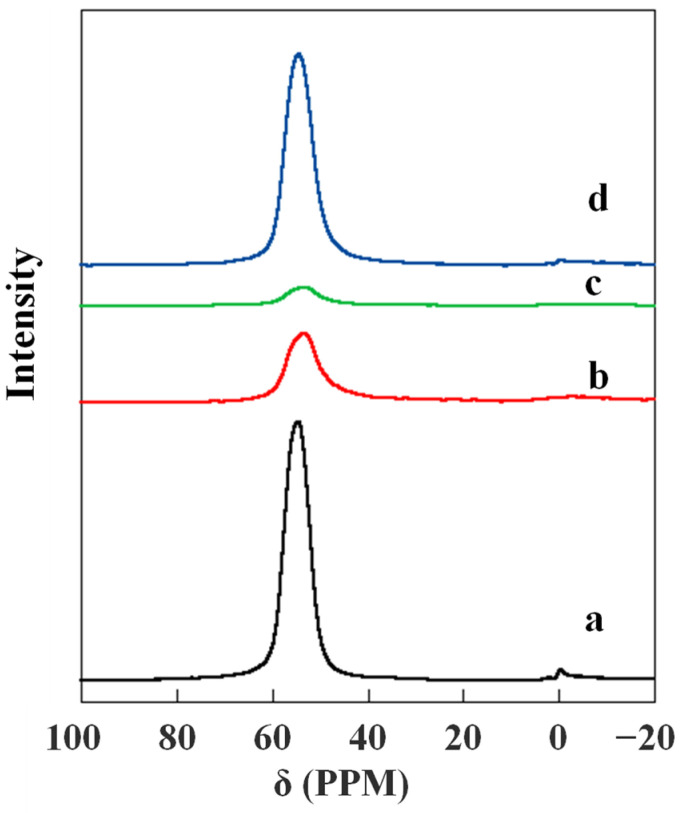
^27^Al MAS NMR spectra of fresh and post-reaction (8 h reaction) H-ZSM-5(80) catalysts. Feed composition: (a) fresh catalyst, (b) ethanol reaction (ethanol: N_2_ = 1:1), (c) ethylene and water reaction (ethylene: water: N_2_ = 1:1:1), (d) ethylene reaction (Ethylene: N_2_ = 1:2).

To confirm the dealumination of the catalysts, the influence of water on the stability of catalytic activity of H-ZSM-5(80) was also investigated. As shown in Figure 7, the propylene yields decreased more quickly with increased water feed composition. Water does not influence the initial activity, but it substantially decreases the structural stability of zeolite. We measured the NH_3_-TPD (Figure S2) and ^27^Al MAS NMR (Figure 8) of all the catalysts before and after reaction. As seen in Figure S2, the catalyst sample exhibited similar NH_3_-TPD spectra with two well-resolved NH_3_ desorption peaks: a low temperature peak around 170 °C and a high temperature peak around 400 °C. The catalyst after 8 h ethylene reaction (Figure S2, spectrum d) exhibited no change compared with the fresh catalyst (Figure S2, spectrum a) before reaction. But for the catalyst after 8 h ethanol reaction (Figure S2, spectrum b), both the high temperature peak and low temperature peak decreased, and the high temperature peak in particular decreased significantly. By way of comparison, the post-reaction catalyst of the ethylene and water reaction exhibited a similar spectrum (Figure S2, spectrum c) with the one obtained after 8 h ethanol reaction (Figure S2, spectrum b). Figure 8 displays the ^27^Al MAS NMR spectra of the catalyst samples. As shown in Figure 8, fresh and post-reaction H-ZSM-5(80) catalysts had vibration peaks at 56 ppm, which indicates that all catalysts contain the same aluminum framework (tetrahedral coordination). However, after the conversion reaction of ethanol, the aluminum content of HZSM-5(80) markedly decreased, with about 38% aluminum content remaining (Figure 8, spectrum b). Although after the conversion reaction of ethylene the aluminum content of the zeolites was still as high as 91% (Figure 8, spectrum d), only approximately 30% aluminum remained on the zeolites after a certain amount of water was added to carry out the reaction (Figure 8, spectrum c). This indicates that the deactivation of zeolites was due to dealumination, and the reason for dealumination was precisely the action of water steam from ethanol dehydration at high temperature.

#### 3.2.5. Contact Time

**Figure 9:**
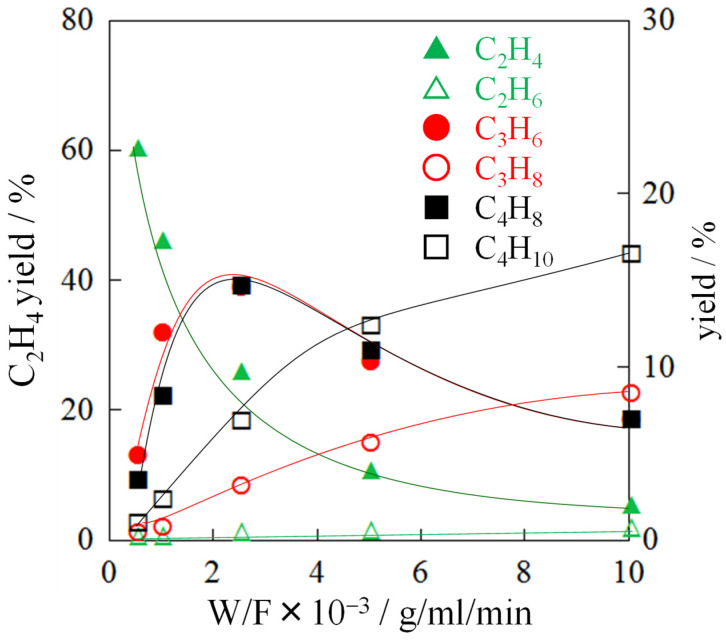
Product distributions of ETO reactions on H-ZSM-5(80) catalyst. Temperature, 400 °C; EtOH concentration, 50%.

The product distribution of ethanol conversions on H-ZSM-5(80) were analyzed under 0.0005, 0.001, 0.0025, 0.005, 0.01 g/cm^3^·min^−1^ (as shown in Figure 9). The ethylene yield decreased as contact time increased. The yields of propylene and butene increased initially, and then decreased as the yields of ethane, propane, and butane increased, which means that the former was hydrogenated to the latter.

On acid catalysts, it appears that ethylene is the main intermediate in the formation of propylene. Busca et al. have shown that, at low temperatures, ether is the first product of the dehydration of ethanol, which can be cleaved to form ethylene [30,31]. Ethanol can also be directly dehydrated in a single molecule to produce ethylene and water at higher temperatures and lower partial pressures of ethanol. Propylene can be produced from ethanol via different routes over acid or base catalysts. Acid catalysts favor dehydration to form ethylene directly or via diethyl ether as an intermediate, then oligomerization to form propylene or higher olefins, followed by metathesis/β-fission to create propylene [3]. The main product selectivity of the H-ZSM-5(50) catalyst in varying reactor space–time was systematically investigated by Busca et al. Ethanol conversion was constant at 100% and ethylene selectivity decreased significantly with increasing contact time, while selectivity for C_3_H_8_, CH_3_CHO and C_5+_ increased slightly. Aromatics did not form at very low W/F (0.0064 kg·h·mol^−1^), but gradually formed as W/F increases [29]. Thus, the relationship of product distribution and contact time in references were similar to our results above.

Based on the above results and reference [3], we propose a catalytic reaction pathway. Ethanol is converted to ethylene by dehydration, and then ethylene is simultaneously converted to propylene, butene, and ≥C5 olefins. Finally, propylene, butenes, and ≥C5 olefins are hydrogenated to propane, butane, and ≥C5 paraffins and aromatics.

#### 3.2.6. Raw Material

**Figure 10:**
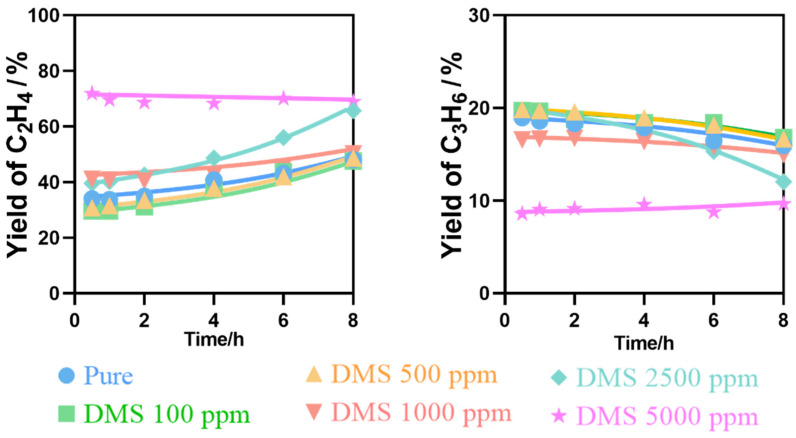
Effect of DMS on catalytic stability of ETO reactions. Temperature, 400 °C; W/F = 0.0025 g/cm^3^·min^−1^.

Alcohols, especially bioethanol obtained by fermentation of plant-derived sugars, starch, and cellulose, are natural fermentation products. Therefore, bioethanol inevitably contains trace quantities of impurities, especially organosulfur compounds such as dimethyl sulfide (DMS) and dimethyl sulfoxide. Organosulfur compounds can cause poisoning of the catalyst used in the reaction. Among them, trace amounts of DMS are usually contained in bioethanol, even after distillation and purification. Therefore, the effect of adding DMS in varying concentrations to ethanol feedstock was investigated. As shown in Figure 10, when the DMS concentration was 100, 500, 1000, 2500 ppm in ethanol, the ethylene and propylene yields were similar to those from pure ethanol transformation. When the DMS concentration reached 5000 ppm, the ethylene yield increased significantly from about 37 to 70%, while the propylene yield dropped significantly from about 22 to 10%.

**Figure 11:**
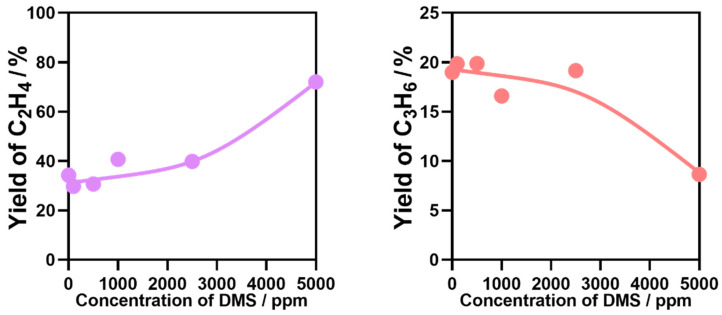
Effect of DMS content on ETO reactions. Temperature, 400 °C; W/F = 0.0025 g/cm^3^·min^−1^.

Figure 11 shows the effect of DMS content on ethylene and propylene yield. It can be seen that the ethylene yield increased and propylene decreased with increasing DMS content. The main reason for these results is that DMS is activated on the surface of zeolite to produce mercaptans. The interaction between H in CH_3_- in mercaptan and nucleophilic oxygen on zeolite weakens the stability of C-H bond, resulting in cracking into olefins, thus the ethylene yield increases. With time on stream, the accumulation of carbon and sulfur on the surface covers some active sites, or combines with a part of the lattice oxygen, which causes the lattice oxygen to be consumed and reduces the catalytic activity. Therefore, the propylene yield decreases. However, due to the small content of DMS, it has little effect on the reaction.

**Figure 12:**
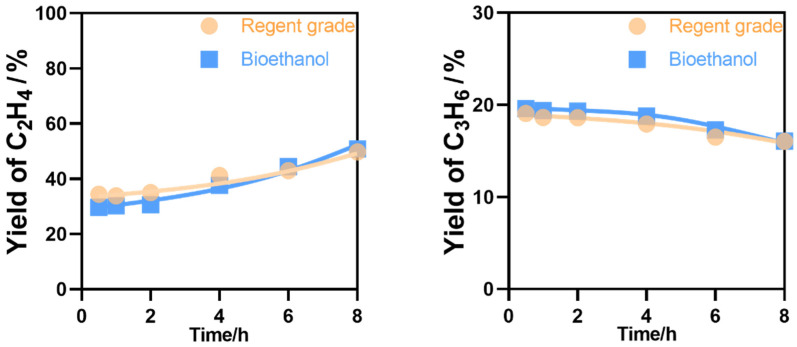
Ethylene and propylene yields of regent-grade ethanol and bioethanol catalytic conversions. Temperature, 400 °C; W/F = 0.0025 g/cm^3^·min^−1^.

Both bioethanol and regent grade ethanol were tested over the HZSM-5 catalyst. As shown in Figure 12, the yield of ethylene and propylene followed a similar trend with time on stream. This shows that the HZSM-5 catalyst is a promising catalyst not only for ethanol but also for the conversion of bioethanol to ethylene and propylene.

## 4. Conclusions

The catalytic reactions of ETP were systematically investigated on different zeolite catalysts. Among the zeolites investigated, H-ZSM-5(80) exhibited the most efficient performance for propylene production. Therefore, the effects of reaction conditions such as reaction temperature and calcination temperature on H-ZSM-5(80) were studied. The moderate acidic sites on ZSM-5 are required for the product of propylene. The activity of ZSM-5 was independent of ethanol feed composition. The H-ZSM-5(80) catalyst was found to be deactivated during ethanol conversion by dealumination, which was caused by water generated from ethanol dehydration. A plausible reaction pathway was proposed. The effects of DMS on ETP reaction were further analyzed, and it was found that DMS did not cause any major deactivation of the H-ZSM-5(80) catalyst. The HZSM-5(80) catalyst is therefore suitable not only for ethanol but also for bioethanol conversion to propylene.

## Data Availability

The data presented in this study are available on request from the corresponding author.

## Supplementary Materials

The following supporting information can be downloaded at: https://www.mdpi.com/article/10.3390/nano12162746/s1, Figure S1: NH_3_-TPD profiles of H-ZSM-5(80) catalysts calcined at different temperatures; Figure S2: NH_3_-TPD profiles of H-ZSM-5(80) catalysts of fresh and post reaction (8 h reaction) H-ZSM-8(80) catalysts.
