# Supplementary material for: Bioethanol Conversion into Propylene over Various Zeolite Catalysts: Reaction Optimization and Catalyst Deactivation
Source: Nanomaterials (Basel). 2022 Aug 10;12(16):2746. doi: 10.3390/nano12162746 (PMC9414363; doi:10.3390/nano12162746)
Supplement: Supplementary file 1 [file nanomaterials-12-02746-s001.zip › nanomaterials-1844702-supplementary.pdf]

Supplementary Information

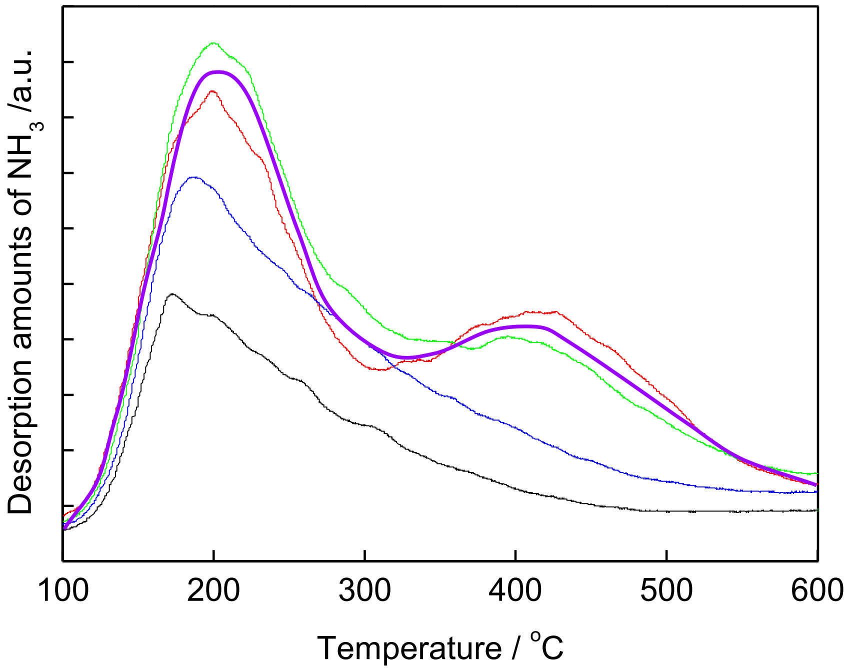

Figure S1. NH_3-TPD profiles of H-ZSM-5(80) catalysts calcined at different temperatures. Calcination temperature: red line 500 °C, purple line 600 °C, green line 700 °C, blue line 800 °C, black line 900 °C.

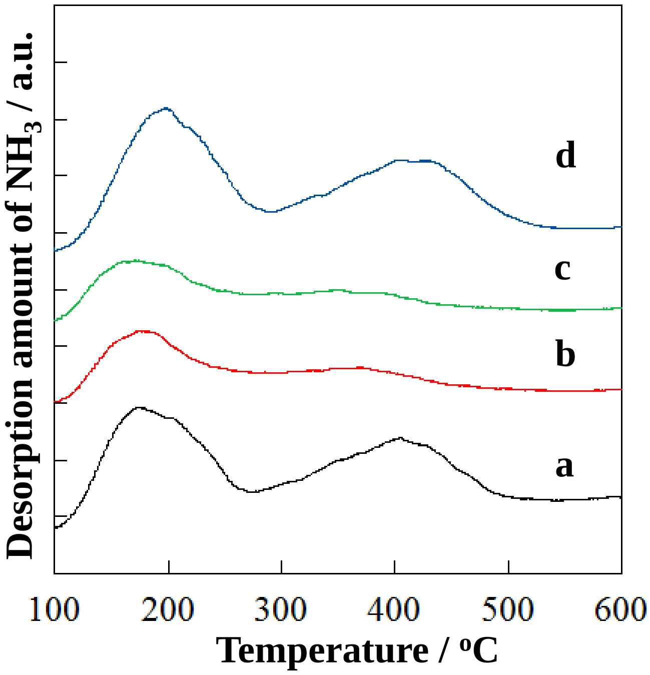

Figure S2. NH_3-TPD profiles of H-ZSM-5(80) catalysts of fresh and post reaction (8 h reaction) H-ZSM-8(80) catalysts. Feed composition: **a**. fresh catalyst; **b**. ethanol reaction (Ethanol: N_2=1:1); **c**. ethylene and water reaction (ethylene : water : N_2=1: 1: 1); **d**. ethylene reaction (Ethylene: N_2=1 : 2).
